# Retrograde intubation in a case of ankylosing spondylitis posted for correction of deformity of spine

**DOI:** 10.4103/1658-354X.62616

**Published:** 2010

**Authors:** Chetankumar Raval, Heena Patel, Pranoti Patel, Utpala Kharod

**Affiliations:** *Assistant Professor, Department of Anesthesia, P. S. Medical College, Karamsad, Gujarat, India*; 1 *Additional Professor, Department of Anesthesia, P. S. Medical College, Karamsad, Gujarat, India*; 2 *Professor and Head, Department of Anesthesia, P. S. Medical College, Karamsad, Gujarat, India*

**Keywords:** *Difficult airway*, *ankylosing spondylitis*, *Retrograde intubation*

## Abstract

Ankylosing spondylitis (AS) patients are most challenging. These patient present the most serious array of intubation and difficult airway imaginable, secondary to decrease or no cervical spine mobility, fixed flexion deformity of thoracolumbar spine and possible temporomandibular joint disease. Sound clinical judgment is critical for timing and selecting the method for airway intervention. The retrograde intubation technique is an important option when fiberoptic bronchoscope is not available, and other method is not applicable for gaining airway access for surgery in prone position. We report a case of AS with fixed flexion deformity of thoracic and thoracolumbar spine, fusion of posterior elements of cervical spine posted for lumbar spinal osteotomy with anticipated difficult intubation. An awake retrograde oral intubation with light sedation and local block is performed.

## INTRODUCTION

Inability to manage difficult airway has been responsible for as many as 30% of the total death attributable to anesthesia.[1] Ankylosing spondylitis (AS) is a form of chronic rheumatic disease that causes arthritis of the spine and sacroiliac joints. It is three times more common in male than female. Approximately 90% of patients are born with the HLAB27 gene. Recently two more genes identified are called ARTS1 and IL23R.[1–3]

The manifestations are limited mouth opening, limited cervical spine movement, atlantoaxial subluxation or fracture. The rigid stiff spine makes positioning very difficult adding difficulty in airway.[2,3] Deranged anatomy and in the absence of fiberoptic bronchoscope, failure of blind nasal intubation and tracheostomy is not practical in this cases, making the retrograde tracheal intubation technique favorable, simple and effectively possible technique.[1,4,5] However, the success rate of the retrograde intubation is variable.[6]

## CASE REPORT

**Figure 1:**
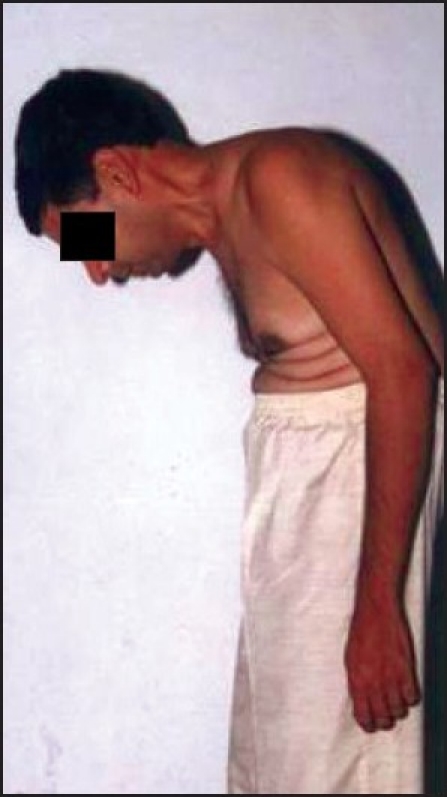
Lateral view of patient's fixed flexion deformity with difficult airway

**Figure 2:**
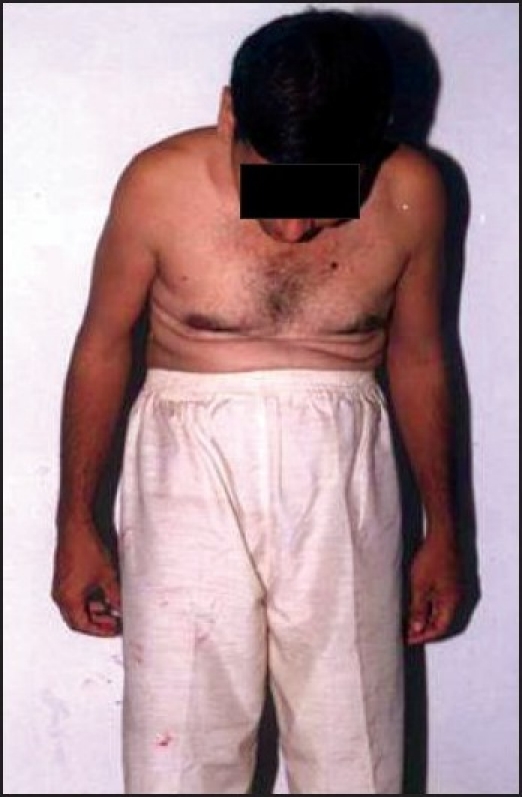
Anterior view of patient's FF deformity with ant. Structure of neck s/o difficult airway

36-year-old male patient, teacher by profession was admitted to Shree Krishna Hospital, for lumbar spine osteotomy with complaints of severe deformity of back with backache, and inability to sleep supine since 10 years. Patient's weight and height was 52 kg and 136 cm, respectively, with BMI: 27.81. Gradually he had increasing deformity of back in the form of forward bending due to which he had difficulty in looking forward. There was no significant medical, neurological, surgical and family history. He was on drug treatment of analgesics, indomethacin and omeprazole [Figures 1 and 2].

On clinical examination, he had a fixed flexion deformity of thoracolumbar region of almost 80 degrees, as confirmed by chin brow angle. This made the patient maintain a constant attitude of extension at the neck in order to achieve a forward horizontal gaze, leading to fusion of posterior elements of cervical spine severely restricting neck movement in extension, flexion and side rotation. Airway examination revealed mouth opening was 4 cm with retrognathia. Mentothyroid distance was less than 6 cm.. On systemic examination there was no limitation of chest expansion and no neurological or cardiac abnormality.

**Figure 3:**
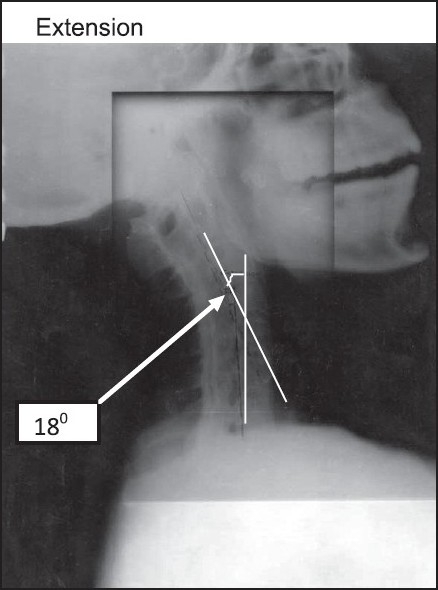
Lateral X‐ray of neck with maximum extension. Maximum extension achieved was 18 degree

**Figure 4:**
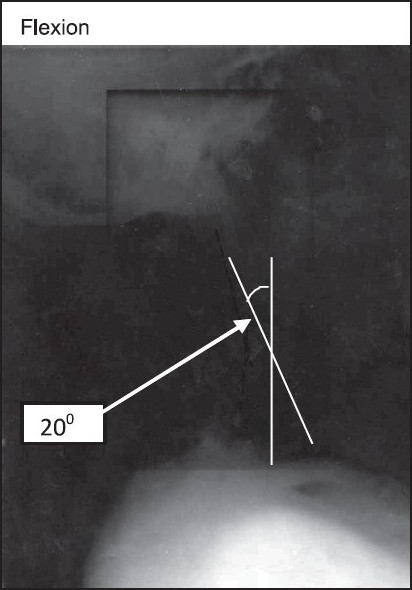
Lateral X-ray of neck with maximum flexion. Maximum flexion achieved was 20 degree

On ENT consultation, nasal passages were normal but vocal cords were not visible on indirect laryngoscopy. All routine and special investigations and pulmonary function tests were within normal. X-ray neck AP and Lateral view showed fusion of posterior element of cervical spine, and maximum extension and flexion was 18 and 20 degrees, respectively [Figures 3 and 4].

Looking at various clinical and radiological deformities, we planned for an awake nasal retrograde intubation with sedation under MAC in the absence of fiberoptic devices. Detail procedure for awake retrograde intubation was explained, and informed consent was taken. Trial of positioning on tipping operation table was given, so that support could be designed to suit the patient′s curvature during intubation. Patient was premedicated with0.2 mg of glycopyrrolate and 30 mg of pentazocine injection IM 45 min before operation. Topical anesthesia and decongestion was achieved by lignocaine 4% and xylometazoline 0.1% nasal pack. Approximately 4% viscous lignocaine gargles was given for oropharyngeal anesthesia. Total lignocaine dose was kept below 4 mg/kg. Routine monitoring with hemodynamicchanges were noted. An ENT surgeon asked to be on standby for possible emergency tracheostomy.

**Figure 5:**
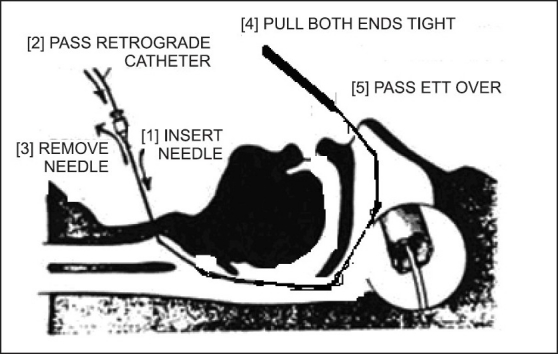
Nasal retrograde intubation

**Figure 6:**
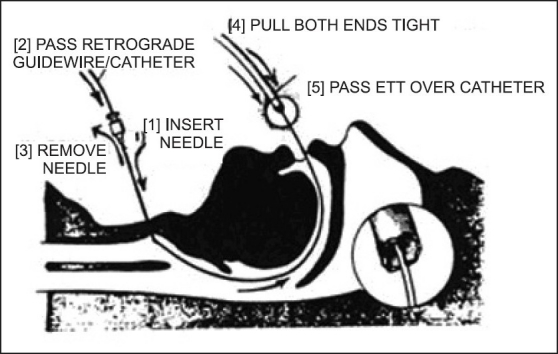
Oral retrograde intubation

**Figure 7:**
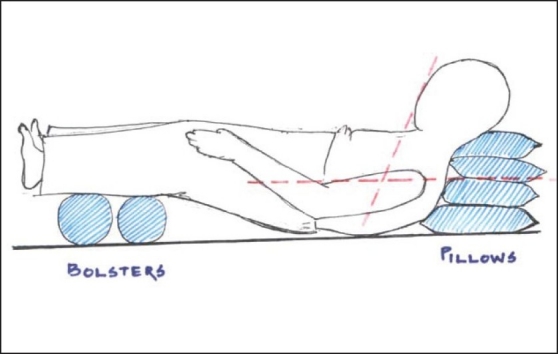
Positioned supine with adequate supports

Adequate supports with four pillows to suit the existing deformity of thoracic and cervical spine and two bolster were placed under the legs to provide a comfortable and for better visualization of larynx. Oxygen was supplemented with nasal prongs. Under aseptic and antiseptic precautions, superior laryngeal nerve block was given with 2.5 mL of 2% lignocaine (plain) after negative aspiration on either side just above thyroid cartilage, at a point 1/3^rd^ distance from midline and tip of superior cornu. Needle advanced 1-2 cm upwards and medially after ′give in′ with great difficulty due to bending forward posture, crowding of all anatomical landmarks in the anterior neck. Cricothyroid membrane was palpated, which was very narrow and infiltrated with1 mL 2% lignocaine. After Local anesthesia 16G epidural needle was passed cranially through the membrane with saline-filled syringe. Tracheal puncture was confirmed by an air aspiration and 1.5 ml of lignocaine 2% was injected. Epidural catheter was passed through the needle. Our plan was to retrieve catheter through the nostril, but due to distorted anatomy it came out through mouth without much difficulty. For nasal intubation, we railroaded the catheter through nostril by red rubber tube. Flexometallic tube 38FG was passed over the catheter; which could not be negotiated into the larynx. Multiple maneuvers like ET tube rotation, catheter tightening-loosening, backward pressure on larynx but failed and nasotracheal intubation was abandoned.[Figure 4–7] The catheter was redirected to oral route and Flexometallic tube 38FG was guided over it. Beveled end of tube was kept facing posteriorly. Lower end of catheter was tightened to guide the tube between the vocal cords, and later it was loosened so that tube could be passed up to desired length. Confirmation of endotracheal tube position was done by auscultation and capnography. Induction of GA was done with propofol 2 mg/kg and atracurium 0.5 mg/kg IV. Anesthesia was maintained with isoflurane and atracurium. Analgesia was supplemented with repeat dose of pentazocine. Smith Peterson osteotomy was performed al L 3-4 level in prone position. Anesthesia was reversed with neostigmine 50 mcg/kg and atropine 20 mcg/kg IV, and extubated in lateral position in the awake state in the OR. Post-operative analgesia was provided with tramadol 1 mg/kg IM 8 hourly. Patient was shifted to high dependency unit for post-operative observation.

## DISCUSSION

Main areas of involvement in AS are sacroiliitis or arthritis, cervical and thoracolumbar spine, pelvis, cricoarytenoid, costovertebral and costotransverse articulations. Temporomandibular joint involvement occurs in 10% of patients, but if disease is long standing, it may increase to 30-40%.[1,2]Sternomanubrium and clavicle joints are also involved. It can be associated with uveitis, ileitis, ulcerative colitis, psoriasis with poor nutrition.[2–7]

Diagnostic criteria : Bilateral sacroiliitis seen on radiograph with one of the followings:

1. Low back pain and stiffness for 3 months′ duration and not relieved by rest.
2. Pain and stiffness of thoracic spine.
3. Limited lumbosacral spine movement in three planes.
4. Limited chest expansion of 2.5 cm, or loss of fourth intercostal space.
5. History or evidence of iritis or its sequelae.[2,3] Treatment may be conservative or operative (lumbar spine osteotomy)[3–7]

Surgery was indicated in our patient for his inability to sleep supine, backache with increasing severity and difficulty in looking forward as he was a teacher by profession. In patient with difficult airway, several techniques are available like blind nasal intubation, retrograde intubation via cricothyroid puncture or fiberoptic intubation.[1]

Awake fiberoptic intubation is a more recent technique, but technically demanding and considered the safest and most effective method in known or suspected cases of difficult airway under direct vision.[8]Though our hospital is tertiary in care level it is not useful due to its unavailability. In the absence of fiberoptic bronchoscope, blind nasal intubation is the method for ET insertion. But due to severe flexion deformity with distorted anatomy, failure of blind nasal intubation is very common.[4,5]And infrequent success of blind intubation is possible at first attempt and increased trauma with repeated attempts, precipitating complete airway obstruction that necessitates emergency cricothyroidotomy, so it was not selected.[9]

Retrograde intubation was originally described by waters in the early 1960s.[9] However it is simple, reliable, applicable method without much difficulty for the teaching purpose of the resident doctors although it is not often used. So we decided to secure airway in our patient with retrograde intubation via cricothyroid membrane with sedation. Applied anatomy of the cricothyroid membrane and retrograde approach has several advantages including absence of bleeding as there are no vessels and fewer chances of subglottic edema and stenosis.[10]

According to availability of the retrograde intubation set, venous catheter, epidural catheter and Seldinger′s wires can be used for retrograde technique.[4,9,11,12]Due to unavailability of retrograde intubation set, we used epidural catheter with epidural needle, which is available everywhere. Multilumen catheter can be used and it allows stabilization and dislodgement of tube at the laryngeal inlet. It can be used for high frequency jet ventilation, providing wider margin of safety.[13]Suction and fluoroscopy can be used for retrieving a catheter or wire from the pharynx,[14,15]and through the nose. A pharyngeal loop can also be used for tracheal intubation in such patient.[5]Route of advancement of tracheal tube over catheter via nasal [13]or oral [16]and attendant complications; many such refinement have been proposed for this method with common goal of achieving an uneventful passage of tracheal tube over retrograde catheter.[5]In our patient we retrieved catheter from mouth without any difficulty and it was railroaded via red rubber catheter through the nose. Hanging up phenomenon occasionally seen, here is also possible in retrograde intubation. It can occur at epiglottis, arytenoids or vocal cords and is overcome by simple maneuvers. [1,8]We had a problem to negotiate tube via nasal route due to distorted anatomy; we tried simple maneuvers but did not succeed. So orotracheal tube was guided over catheter. Maximum allowable dose of lignocaine is 4.5 mg/kg [17]Patient was hemodynamically stable except transient tachycardia, increase in SBP [+ 15% of baseline] and co-operated well during the procedure, which took12 min. Post-operatively patient was shifted to HDU for observation where he was stable except for the soreness of throat and mild leak of CSF initially.

## CONCLUSION

Death from loss of airway still occurs in patients with difficult airway. Data suggest that in the absence of fiberoptic devices, retrograde approach via catheter is the safe, simple and effective technique. We could successfully manage this case of extremely difficult positioning and difficult airway with awake oral retrograde intubation under sedation.
